# Ethics as attention to context: recommendations for the ethics of artificial intelligence

**DOI:** 10.12688/openreseurope.13260.2

**Published:** 2021-12-13

**Authors:** Anais Resseguier, Rowena Rodrigues

**Affiliations:** 1 Trilateral Research, Waterford, X91 W0XW, Ireland; 2 Trilateral Research, London, SW1X 7QA, UK

**Keywords:** AI ethics, ethics as attention, practical ethics, social impacts of AI, technology ethics, social sciences and humanities, technology and society

## Abstract

This article shows that current ethics guidance documents and initiatives for artificial intelligence (AI) tend to be dominated by a principled approach to ethics. Although this brings value to the field, it also entails some risks, especially in relation to the abstraction of this form of ethics that makes it poorly equipped to engage with and address deep socio-political issues and the material impacts of AI. This is particularly problematic considering the risk for AI to further entrench already existing social inequalities and injustices and contribute to environmental damage. To respond to this challenge posed by AI ethics today, this article proposes to complement the existing principled approach with an approach to ethics as attention to context and relations. It does so by drawing from alternative ethical theories to the dominant principled one, especially the ethics of care or other feminist approaches to ethics. Related to this, it encourages the inclusion of social sciences and humanities in the development, deployment and use of AI, as well as in AI ethics discussions and initiatives. This article presents this proposal for an ethics as attention to context and formulates a series of practical recommendations to implement this proposal concretely.

## Introduction

The ethics of artificial intelligence (AI) have generated a large amount of interest over the last few years ^1^. The numerous ethics guidelines and other forms of ethics initiatives produced provide ample evidence of this ^2^. Although the field has seen major developments in various arenas (including in academia, industry, and policy), it has also come under intense criticism for being too abstract and high-level, and therefore, unable to properly guide technological development, deployment and use ^3^. Critics have highlighted that some of these initiatives lead to ethics washing ^4^ and/or contribute to reproducing structural inequalities in society ^5^.

Although this essay recognises the value of the ethics of AI and what they have produced over the last few years, it also acknowledges that efforts in this area are still needed, especially *to move beyond the abstraction of current AI ethics initiatives*. More recently, there have been numerous efforts at making ethics guidelines more fit for purpose by operationalising them to specific sectors of application or development contexts ^6^. These are assuredly necessary and praiseworthy efforts.

Several experts on AI have critiqued AI ethics for failing to consider contextual elements, especially the socio-political context, which, as they note, is essential to address ethical challenges posed by this technology ^7^. This essay shows that this identified gap in AI ethics finds its root in the very nature of the currently dominant approach to AI ethics, i.e., *a view on ethics that considers it as a softer version of the law*. It points to the need to complement this approach and makes a series of practical recommendations. As such, it calls for *a shift of attention in AI ethics: away from high-level abstract principles to concrete practice, context and social, political, environmental materialities.* It draws extensively from critical approaches to AI and ethics, especially those emerging from feminist perspectives (including the ethics of care). It also derives from consultation with a number of stakeholders as part of SIENNA engagement ^8^.

This essay first undertakes a theoretical detour to better understand the nature of the ethics approach at stake in current AI ethics (section 2) and how to best complement it (section 3). This detour is necessary to build the conceptional groundwork for the practical recommendations developed in section 4, which contains the actual proposals to promote ethical AI. This essay, and the guidance it offers, are addressed to policymakers in government, AI ethicists, engineers (software engineers, data scientists, etc.), organisations developing AI, and, more generally, anyone concerned by the development, deployment and use of AI and the potential social and ethical impacts of this technology ^9^.

## Current AI ethics and the risk of abstraction

### The principled approach to ethics in AI ethics

The numerous AI ethics initiatives, documents and guidelines produced over the past few years have primarily taken the shape of high-level abstract and prescriptive principles, such as “Ethics guidelines for trustworthy AI” of the High-Level Expert Group on AI (AI HLEG) set up by the European Commission, the “Recommendation of the Council on Artificial Intelligence” by the Organisation for Economic Co-operation and Development (OECD), or on the industry side, the Google AI principles ^10^. However, as Resseguier and Rodrigues have argued, these AI ethics initiatives are dominated by a principled approach, i.e., a “law-conception of ethics”, which is a view on ethics that considers it as a *replica* of the law, i.e. a softer version of the law ^11^. Resseguier and Rodrigues point to the risk of misusing ethics as a replacement for legal regulation, and in particular the risk of ethics washing. While legal regulation might come with hard lines that could restrict innovation, regulation through ethical principles and guidelines offer more flexibility and leeway; hence, they are favoured by industry actors ^12^. An issue with this principled approach to ethics is that it leads to ethics being used as a weaker form of regulation by actors with interest in avoiding hard lines ^13^. Additionally, this approach to ethics comes with another pitfall that needs to be addressed: *its abstraction and the risk of disconnection from social, political and environmental materialities*.

The ethical theory of the “ethics of care” or “care ethics” that emerged in the 1980s with the work of Carol Gilligan has identified a fundamental gap in the principled approach to ethics, a conception that the ethics of care has called “ethics of justice” or “principlism”: its neglect of context and actual practices, i.e. its abstraction ^14^. This neglect is not simply a side-effect of this approach; *it is one of its constitutive features*. Indeed, as the ethics of care shows, the principled view on ethics is primarily and fundamentally characterised by its gesture of abstraction from concrete situations. It is through this abstraction that it can develop the general and impartial principles that it relies on and seeks to promote. This is particularly clear in the “veil of ignorance” promoted in the ethics of John Rawls. This veil aims at hiding any elements that constitute a person’s specific socio-political situation in the world (including race, gender, socio-economic status, nationality, etc.) in order to formulate judgements from an unbiased and impartial standpoint, what Thomas Nagel has called the “view from nowhere” ^15^. Although this approach to ethics has its value, its abstraction also brings with it several blind spots that are particularly problematical in the context of AI, even more so when it is applied to a field such as AI which presents itself as supposedly immaterial.

### AI immaterial narrative and AI ethics

There is a dominant narrative surrounding digital technologies that presents these technologies as intangible. The supposed dematerialisation of technology is a narrative that has existed before digitalisation; however, it has become particularly pervasive with digitalisation, and especially AI ^16^. A telling example of this is the concept of the “cloud” that makes data storage appear as deprived of a physical existence and, therefore, of practical impacts on the society and the environment. However, as we know, this is far from being true. The “cloud” relies on giant data centres that consume massive amount of energy. Similarly, studies have shown that the training of AI systems requires a high level of energy consumption as well as resource extraction, both of which have significant environmental costs ^17^.

The situation of micro-workers in the AI ecosystem today provides another clear case that undermines the AI “immaterial narrative”. Micro-workers are people who work for platforms, such as Amazon’s Mechanical Turk, to prepare, label and verify the data that go into AI systems. The AI industry tends to hide the situation of these workers, pretending that all the hard work is fully automatised. However, this is fallacious: AI systems rely on the work of people around the world that are extremely poorly paid and are working with no social security protection ^18^. Here as well, AI “immaterial narrative” hides highly problematical impacts on the society, in this case those related to working conditions and employment rights.

Hence, this “immaterial narrative” that serves to pretend that AI, because intangible, is deprived of questionable social or environmental impacts, is highly problematic. However, because of its neglect of situatedness and materialities to reach high-level abstract principles, the dominant approach in AI ethics today renders it ineffective at properly addressing these negative material implications of AI. In other words, the disconnected nature of AI ethics today does not allow an appropriate response to some of the key critical issues that AI raises for society and individuals ^19^. It is therefore necessary to complement it with methods to ensure AI ethics turns to concrete practices, considers the socio-political context and materialities, and addresses impacts.

### AI ethics and the neglect of structural inequalities

This need for AI ethics to engage with the socio-political reality is even more necessary to address a major issue of AI: the risk of this technology further entrenching already existing structural inequalities. As Klein and D’Ignazio have shown in *Data Feminism,* AI ethics at present appear inadequate to properly respond to this challenge. According to them, data ethics is a field that relies on “concepts that secure power”, and that, as such, “maintain the current structure of power” ^20^. This is highly problematic as it renders AI ethics unable to address the root causes of one of the most significant ethical issues of AI: biases and social inequalities, especially those pertaining to race and gender. Studies have shown that the most vulnerable populations are those who face the most problematic impacts of AI (and often who are subject to the deployment of AI technology without choice or ability to influence its design and development), while, on the contrary, those who benefit the most from AI are those who hold positions of power in relation to this technology ^21^. For instance, the study “Gender Shades” shows this clearly, demonstrating the problematic consequences of producing facial recognition systems trained with white males as the norm ^22^. The consequence is a technology that works best for white males, but poorly for black females, white females and black males lying in between. Here as well, the “point of view of nowhere” that characterises AI and AI ethics fails to offer a proper response to key issues in the field of AI, particularly those related to structural inequalities and injustice ^23^. Hence, AI ethics needs to be complemented in a way that enables it to address and respond to these issues.

There is an interesting parallel to be drawn between (1) the criticism toward AI ethics as being elaborated from the perspective of the privileged members of society and (2) the criticism formulated by the ethics of care toward what it identified as the dominant form of ethics (the ethics of justice). On the side of the critique toward AI ethics, Klein and D’Ignazio point to the “privilege hazard” which they define as “the phenomenon that makes those who occupy the most privileged positions among us—those with good educations, respected credentials, and professional accolades—so poorly equipped to recognize instances of oppression in the world”, i.e. an “ignorance of being on top” ^24^. The ethics of care highlights a similar situation characterised by the “indifference” of those who are privileged, i.e. those who occupy positions of power ^25^.

Thus, the dominant form of ethics in current AI ethics is *primarily a principled one characterised by abstraction*. This approach to ethics makes AI ethics *poorly equipped to respond to some of the key issues of AI related to social, political and environmental materialities*. Therefore, the ethics of AI as it has been developed over the past few years *needs to be complemented by other means to ensure proper consideration of actual practices, situations and socio-political context*.

## For an ethics of attention to context, situatedness and materialities

The second section pointed to one of the key risks of the dominant understanding of ethics in current AI ethics discourses and initiatives: its abstraction leading to a potential neglect of problematical social, political and environmental impacts of AI. The third section presents how this approach can be best complemented to respond to the identified limitation. It does so by promoting a different approach to ethics, one that *invites to a sharp attention to context, situatedness and materialities*. This section also responds to feedback from SIENNA’s stakeholders and other AI experts who have repeatedly called for making AI ethics more practical and usable for organisations and developers ^26^.

### Ethics as attention in situation

This section argues for the *need for AI ethics to shift attention away from high level abstract and prescriptive principles to practical contexts and relations*. This shift is one of the lessons learned in bioethics, a field that was at first heavily dominated by high level principles, such as in the famous *Principles of Bioethics* by Beauchamp and Childress that derive answers to ethics challenges from four basic principles (autonomy, beneficence, non-maleficence, and justice). This approach has been challenged for being too abstract, top-down and insufficiently attentive to particulars ^27^. Aren’t we reproducing the same issue in AI ethics today as bioethics in the 1980s with the dominating principled approach (or “principlism”)? As indicated by a participant to the SIENNA workshop on multi-stakeholder strategies for ethical AI (Sept 2020), AI ethics should draw from lessons learned in bioethics ^28^.

The ethics of care has developed resources to move beyond the principled approach to ethics. It has called for ethics to “modify its field: from an enquiry into general concepts to the study of particular situations, individual’s moral configurations” ^29^. It moves away from the “view from nowhere” that characterises the principled approach and invites to a sharp attention to concrete practical reality, situations and relations.

This attention to specific situations is more generally a key contribution from feminist theory. As Klein and D’Ignazio put it: “one of the central tenets of feminist thinking is that all knowledge is situated” ^30^. The recognition of the situatedness of knowledge is a particularly essential recognition for the fields of AI and data science, fields that are often “framed as an abstract and technical pursuit” and that, as such, leave aside the “social context, ethics, values, or politics of data” ^31^. Data science has the tendency to pretend it is always the same regardless of its context of application, whether it is astrophysics, criminal justice or carbon emission ^32^. This is deceptive and the ethics of AI needs to be able to challenge and address this fallacious claim. To do so, it needs to advocate for attention to social, political, environmental context and materialities ^33^.

For instance, when dealing with criminal data, it is essential for AI ethics to recall the historical structures of injustices and inequalities through which these data have been produced ^34^. Similarly, when handling health data, one should have in mind that white males are significantly over-represented in such sets of data, leading to the risk of poor quality healthcare for women and the non-white population ^35^. The case of the machine learning technique of word embedding is another telling example of the need to consider the social background, as Bolukbasi *et al*. show clearly in their article “Man is to Computer Programmer as Woman is to Homemaker?” ^36^

Ethics defined as attention means attention to socio-political realities and materialities at, at least, two different levels: (1) that of the development of an AI system (both from the production of the algorithm and the datasets that were used for the training of the algorithm) and (2) that of the context of application and intended use. The proposal formulated by Gebru *et al.* documenting the “motivation, composition, collection process, recommended uses, and so on” of a database used for machine learning would be particularly useful in that regard ^37^. Another expression of ethics as attention to context is developed by Asaro in an article in which he calls for an ethics of care approach to AI ethics in predicting policing. He highlights the need for AI ethics “to seek likely ways in which political, economic, or social pressures may have influenced historical datasets, to consider how it [sic] may be shaping current data collection practices, and to be sensitive to the ways in which new data practices may transform social practices and how that relates to the communities and individuals a system aims to care for” ^38^. To do so, he recommends the inclusion of “domain experts as well as critical social scientists as members of design teams” and the recognition of the “necessity of their expertise in shaping ultimate system design” ^39^.

### Ethics as attention to relations of power

Another crucial insight from the ethics of care that AI ethics can draw on is the need to pay closer attention to *relations of power and inequalities*. When the ethics of care argues that ethics is a matter of attention to situations, this includes relations. It is important to note that these relations do not only refer to interpersonal relationships, although these are at stake as well. It also refers to broader relations in the society, including relations of power, i.e. power asymmetries between different social groups. This is one of the key contributions of the ethics of care to ethical theory. It is also a call for realism. As Laugier puts it: “Care takes us back to this requirement of realism in the sense of the need to see what is in front of our eyes: the reality of inequality before the idealness of principles” ^40^. This is particularly essential in the context of AI considering the high economic interests involved and how these come to shape policy agenda on the regulation of AI.

Several experts have shown that the dominant form of ethics has failed to pay sufficient attention to the power imbalance at stake in the discussions on the regulation of AI, especially with regards to the concentration of power in the hands of a few big tech companies. For instance, Wagner shows how ethics has been used “as an escape from regulation” ^41^. Article 19, a non-governmental organisation, argues that ethics initiatives have often “proven to be a strategy of simply buying time to profit from and experiment on societies and people, in dangerous and irreversible ways.”

In the face of this, it is essential for ethicists to (1) avoid developing tools and resources that may serve this form of misuse of ethics, and (2) combat these misuses. To do so, ethicists need to be clear about the power relations and economic interests at stake. For instance, with these interests at stake, they need to consider when self-regulation is a meaningful and potentially effective response and when it is not. For instance, Access Now, another non-governmental organisation, has demonstrated in a recent report, “taking an ‘ethics’-based approach to facial recognition and other dangerous applications of AI would leave millions exposed to potential human rights violations, and with little to no recourse.” As a SIENNA stakeholder put it: we need to be clear on what we can realistically expect from ethics. This is even more important bearing in mind the power of the big technology companies, especially GAFAM (Google, Apple, Facebook, Amazon, and Microsoft) ^42^.

Paying attention to relations of power in the AI field also entails considering the severe diversity issue in the AI community. The 2019 report by the AI Now Institute “Discriminating Systems. Gender, Race, and Power in AI” powerfully points to this issue and calls on the AI industry to “acknowledge the gravity of its diversity problem” ^43^. This “diversity problem” is not only an employment issue; it has direct implications on the type of AI systems produced. As West *et al.* write, “these patterns of discrimination and exclusion reverberate well beyond the workplace into the wider world”, i.e. they lead to the development of technological products that are more beneficial to males than females or non-binary, to white rather than non-white ^44^. Once again, this is essential to take into account for an AI ethics that directly engages with its situatedness and develops adequate tools to ensure AI systems are assessed with their social, political, and environmental impacts in consideration, so that the negative ones may be properly addressed and mitigated.

### Enabling ethical agency

Hence, this essay argues that ethics must entail a sharp attention to specific situations and relations, accounting for the different levels of the personal, the interpersonal, the organisational, up to broader social, political, and environmental configurations. But a question then arises: how can there be any ethics guidance, if ethics is primarily a matter of attention to specific situations? In other words, isn’t this approach to ethics as attention rendering any recommendations inadequate as they would take away from the specificities of the context? Although this essay argues that ethics is primarily a matter of attention to specific situations, guidance is still needed. However, the status of such ethics guidance needs to be specified (the guidance formulated in this piece is precisely of this nature).

The recommendations formulated here do not aim to say what one should do or should not do, but rather aim to *promote the conditions of possibility of doing ethics, i.e., of being able to identify the right course of action from within a particular situation*. In that sense, the form of ethics that is promoted here does not determine the ‘right’ or the ‘good’ from a distanced position; it is not prescriptive or imperative. Rather, it aims to provide tools and resources to ensure actors, in their situated position – such as a developer in an organisation, within her own role, position, organisation, socio-political-environmental context, and confronted with a particular engineering challenge – can make ethical choices. Rather than determining from a distanced position what the ethical thing to do is, it should be ensured *actors are in a position to determine what is the right thing to do*. To use the terminology proposed by Canguilhem, it is not a matter of determining norms, i.e. the right thing to do, but of developing, actors’ “ *normative capacity*”, which we can also define as ethical agency ^45^.

This approach avoids the risk of ethics guidance being patronising. This is one issue that was raised by a SIENNA stakeholder from a big technology company: the risk of the ethicist “coming on high horse” with predetermined ideas of what engineers should do. As Miller and Coldicutt show, technology workers are sensitive to the impacts of their products: “79% agree it’s important to consider potential consequences for people and society when designing new technologies” ^46^. They have called this capacity “personal moral compass” ^47^. For AI ethics, it is essential to recognise this already existing sensitivity to issues and ability to respond to ethical challenges. However, as participants in the SIENNA workshop on multi-stakeholder strategies for ethical AI highlighted, this is not sufficient. This ethical sensitivity and ability need to be further enhanced, promoted and protected. This is precisely the objective of the practical recommendations below. Promotion and protection of ethical sensitivity and ability can take various shapes. One of these is by conducting *impact assessment* of AI technologies and products. Miller and Coldicutt point to the fact that 81% of people in AI “would like more opportunities to assess the potential impacts of their products” ^48^. These can also take the shape of operationalised guidance documents, such as those developed in SIENNA with the ethics by design methodology, or research ethics guidance documents.

It is essential to clarify that these are only tools to promote ethical sensitivity and the ability to make the right decision. Human expertise and sense of the specificity of the situation at stake (e.g. *this* AI system developed in such and such context with such and such objective) remains essential. In other words, high-level principles, norms and guidance, although they can help provide the broad lines, cannot fully dictate what should be done and what should not be in a specific situation. There is always, necessarily, the need to pay attention to the specificities of a particular situation. Therefore, for instance, in addition to research ethics guidelines, research ethics also needs human expertise in order to ensure the guidelines (necessarily general) are properly applied and interpreted with a specific situation (necessarily particular). The EUREC ^49^/SIENNA online workshop on 26–27 October 2020 that brought together members of European research ethics committees and SIENNA consortium partners on the topic of research ethics guidelines made this clear: expertise in interpreting and applying ethics guidelines to specific research cases is essential to research ethics ^50^.

Another way of ensuring AI products/technologies are developed with care for their socio-politico-environmental impacts, is through the *protection of whistle-blowers and unions*. The need to protect whistle-blowers was mentioned by a participant in the Multi-Stakeholder Strategies for Ethical AI workshop on 8–9 September 2020. The protests by Google employees against the Maven project, in which Google was developing AI technology for the US military drone programme, is a strong example of this. Google employees saw issues with a powerful technology company such as Google getting into “the business of war” ^51^. Considering ethics implies asking hard questions, and those who have the courage to do so should be protected. In other words, if we want workers “to be vectors of change”, they need to be able to raise concerns when they see something that is problematic in their organisation and be protected appropriately from the ensuring fall out.

## Conclusion

To conclude, this essay has shown that current AI ethics guidance and initiatives tend to be dominated by a principled approach to ethics. Although this brings value to the field, it also entails some risks, especially in relation to the abstraction of this form of ethics that makes it poorly equipped to engage with and address deep socio-political issues and practical impacts. As such this essay has sought to complement the existing principled approach to ethics with an approach to ethics as attention to context and relations. Below are some more practical recommendations to promote ethical AI by drawing from an approach to ethics as attention ^52^.

## Practical recommendations for AI ethics

*AI ethicists should:*

→ **Engage with social scientists and their research on social impacts of AI** in the short, medium and long term
→ **Engage with data scientists and software engineers** to better understand the way AI systems are developed and the data collected, cleaned, processed and interpreted
→ **Draw from ethics theories beyond principlism**, especially the ethics of care, virtue ethics, or Spinozist ethics.
→ **Ensure diversity** in the composition of AI ethics teams (especially encouraging inclusion of non-white females and non-binary individuals).
→ **Pay attention to the “privilege hazard”**, i.e. the risk of people in position of privilege failing to notice instances of oppression and injustices perpetuated by AI technologies
→ **Recognise, build upon and further develop AI developers’ ethical sensitivity** (rather than impose guidance that may be top-down and disconnected from practice)
→ **Develop/share use cases on ethical and social impacts of AI** (especially negative ones) to make the impacts of AI more concrete and understandable
→ **Engage more with the impacted communities, especially the most vulnerable among them,** and consider social and ethical impacts from their perspective
→ Take into consideration the **environmental impacts of AI** (throughout the whole life cycle, from resource extraction to end-of-life disposal)
→ When carrying out impact assessments, consider **impacts on labour**, especially the conditions of “micro-workers” and other precarious workers in the AI industry

*The AI industry should:*

→ Engage with **social science studies** on the social and material impacts of AI
→ Promote **inclusion of social scientists** in AI research and development projects
→ Recognise that a technological product is **never “neutral”** and always reflects specific worldviews and intentions
→ Conduct/require **assessments of the ethical, social, environmental, and human rights impacts of AI** before, during and after deployment of AI systems
→ **Encourage prudence and honesty about the capabilities of the AI system being sold and do not gloss over the limitations**
→ **Ensure diversity** in the composition of the team researching, developing, using, or assessing AI (especially encouraging inclusion of non-white females and non-binary).
→ **Pay attention to the “privilege hazard”**, i.e. the risk of people in position of privilege failing to notice instances of oppression and injustices perpetuated by AI technologies
→ **Provide a safe environment for whistle-blowers and union members** in the AI industry
→ Develop and ensure respect for **research ethics in the private research and development (R&D) AI sector.**
→ Promote and create r **esearch ethics committees/AI ethics officers** for AI R&D in the private sector
→ **Engage more with impacted communities, especially the most vulnerable among them,** and consider social and ethical impacts from their perspective
→ **Recognise that not all social problems can be solved with technology**, i.e. be wary of technosolutionism

*AI researchers and developers should:*

→ Engage with **social science studies** on the social and material impacts of AI
→ Promote the **inclusion of social scientists** in AI projects
→ Recognise that a technological product is **never “neutral”** and always reflects specific worldviews and intentions
→ Conduct/require **assessments of the ethical, social, environmental, and human rights impacts of AI** before, during and after deployment
→ **Consider the possibility of *not* developing an AI system** when the ethical and social issues identified are too severe and difficult to mitigate. Also account for the remaining uncertainties surrounding the ethical and social impacts of AI.
→ **Ensure diversity** in the composition of the team developing, using or assessing AI (especially encouraging inclusion of non-white females and non-binary individuals).
→ **Pay attention to the “privilege hazard”**, i.e. the risk for people in position of privilege to fail to notice instances of oppression and injustices perpetuated by AI technologies
→ **Report on the development of AI technologies which are harmful to society and violate human rights,** and report incidents to AI incident databases and regulators or civil society organisations, particularly where no impact assessment of such technologies has taken place.
→ **Engage more with the impacted communities, especially the most vulnerable among them,** and consider social and ethical impacts from their perspective
→ **Recognise that not all social problems can be solved with technology**, i.e. be wary of technosolutionism

*Policy-makers engaged in AI regulation and governance should:*

→ **Not let the AI hype hide the ethical and social issues this technology brings about and the difficulty to solve them** (e.g. exacerbation of structural inequalities, privacy risks and surveillance) as well as the remaining uncertainties on the ethical and social impacts of AI
→ **Protect whistle-blowers and unions** in the AI industry and develop adequate protection mechanisms and safeguards where this is missing.
→ **Encourage** **research ethics in private-sector AI R&D**
→ **Encourage the** **creation/use of research ethics committees** for AI R&D in the private sector
→ Require/encourage/mandate the conduct of **assessments of the ethical, social, environmental, and human rights impacts of AI** before, during and after deployment.
→ **Engage more with the impacted communities, especially the most vulnerable among them,**and consider social and ethical impacts from their perspective
→ **Recognise and promote awareness of the fact that not all social problems can be solved with technology**, i.e. be wary of technosolutionism
→ **Be wary of the AI hype**

*AI public research funding organisations should:*

→ **Fund cutting-edge social science studies** on AI and its impacts in the short, medium, and long term.
→ Require/encourage the conduct of **assessments of the ethical, social, environmental, and human rights impacts of AI** research and development
→ **Engage more with impacted communities, especially the most vulnerable among them,** and consider social and ethical impacts from their perspective
→ **Recognise and promote awareness of the fact that not all social problems can be solved with technology**, i.e. be wary of technosolutionism
→ **Be wary of the AI hype**

*AI STEM (science, technology, engineering, mathematics) educators should:*

→ **Train AI researchers and developers** on the non-neutrality of AI and familiarise them with AI ethical and social impacts and impacts on human rights
→ **Develop/share use cases on the ethical and social impacts of AI** (especially negative ones) to make the impacts of AI more concrete and understandable
→ **Recognise and promote awareness of the fact that not all social problems can be solved with technology**, i.e. be wary of technosolutionism

## Data availability

No data are associated with this article.

