# Supplementary material for: Ethics as attention to context: recommendations for the ethics of artificial intelligence
Source: Open Res Eur. 2021 Dec 13;1:27. Originally published 2021 Mar 26. [Version 2] doi: 10.12688/openreseurope.13260.2 (PMC10446024; doi:10.12688/openreseurope.13260.2)
Supplement: Peer review history

# Peer review: Reviewer response for version 1

**DOI:** 10.21956/openreseurope.14330.r27581

**Published:** 2021-10-06

**Authors:** Mathieu Corteel

**Affiliations:** 1 Laboratoire interdisciplinaire d'évaluation des politiques publiques (LIEPP), Sciences Po, Paris, France

The article develops a materialist perspective to consider the socio-political relations through the ethics of Artificial Intelligence (AI). Against the ideological discourses of AI promoters (immaterial narrative), the authors adopt a realist position based on attention to context.

 They emphasize the fact that the ethics of AI is always to be remade according to social, political and environmental aspects. In order to improve the ethics of AI, they suggest shifting from a top-down ideological approach to a bottom-up materialist approach.

 The main merit of the article is therefore to highlight the ineffectiveness of abstract "ethical" principles promoted by AI industry. However, the article takes the form of a statement of intent rather than a useful tool for implementing a new AI ethics.

1. The exhaustive list of “practical recommendations”, at the end of the article, suffers from the same defect as abstract and formal theories of ethics: it proposes a list of moral precepts and recommendations (ensure diversity, inclusion of social scientist, take into consideration the environmental impact...) without methods of implementation. The authors are, in a way, victims of what they denounce.
2. The ethics of care is invoked as a guarantor of effectiveness against discrimination and concentration of power, but it is not explicitly detailed as a pragmatic way to adapt AI ethics to concrete contexts.
3. There is no specific evidence in the article demonstrating the relevance of materialist ethics or ethics of care to improving the practices of data science, data-driven decision making, AI design or social applications of AI. How do we make ethics an implemented capability in the practice of citizens, engineers and organizations developing and using AI?

In order to improve the article, I suggest that the authors:

1. Explain more specifically how to implement the “practical recommendations” through effective ethical capabilities in the design and use of AI. This includes case studies that demonstrate the relevance of these recommendations to ethically improving decision and action in context.
2. Detail the pragmatic basis and method of their materialist perspective by considering what the practice of ethics is in an AI application setting.
3. Clarify the need for interdisciplinarity to improve the design and adaptation of AI to diverse social and cultural contexts.
4. Indicate which disciplines and methods are most relevant to participate in this heuristic innovation.

Is the argument persuasive and supported by appropriate evidence?

No

Is the work clearly and cogently presented?

Yes

Is the topic of the essay discussed accurately in the context of the current literature?

Yes

Does the essay contribute to the cultural, historical, social understanding of the field?

Partly

Reviewer Expertise:

Philosophy of Science, History of Science, Medical Ethics, Ethics of Technology

I confirm that I have read this submission and believe that I have an appropriate level of expertise to confirm that it is of an acceptable scientific standard, however I have significant reservations, as outlined above.

# Response

**Published:** 2021-11-26

**Authors:** Anais Resseguier

Many thanks for the detailed review and comments. Please find below a response to each of them. On the first 3 comments:

1. There might be some confusion regarding the scope of this article. We added a note on Endnote 50 to clarify and hope that this helps sort this out.
2. We are not sure what might have given the impression that we ‘invoke’ the ethics of care as ‘a guarantor of effectiveness against discrimination and concentration of power’. The contribution of the ethics of care here is that it makes it possible to approach the ethics of AI differently from the currently dominating approach which is characterised by “principlism”. The ethics of care gives theoretical tools to challenge this dominant approach and to develop a new approach (new approach that is then complemented by more practical recommendations). As indicated in the paper, we see the ethics of care as having ‘developed resources to move beyond the principled approach to ethics’, and the ‘view from nowhere’ that characterizes this approach. We see value in the ethics of care as it invites to ‘pay closer attention to *relations of power and inequalities*.’
3. This comment might relate to the overall objective and scope of this article. The main objective of this article is twofold: 1. Critically present the current landscape of AI ethics and where/how we think it might be failing/ineffective (this is done in 1 ^st^ section). 2. Provide a different approach to thinking about the ethics of AI (through ethics as attention to context) (this is done in the 2 ^nd^ section). The Practical recommendations at the end highlight some practical steps to operationalise this different approach to ethics. Hence, we believe we started looking into the implementation of this approach in this last section; however, it is not the core objective of this article and is therefore not developed further. Developing this further would require another article, that could draw on the theoretical foundations laid down in the present article.

Regarding the suggestions to improve the article:   Points 1 and 2: Here too, we see this as being outside the scope of this article. We trust the responses above will help clarify the scope. A case study would certainly be highly valuable but falls outside the scope of the present article. Points 3 and 4: We are slightly unsure what the suggestions are about and what they try to achieve. The article is interdisciplinary and draws from philosophy, ethics and social sciences (as made obvious in this paper). It also draws from discussions with experts coming different disciplines consulted as part of the SIENNA Horizon 2020 project, as explicitly stated in the paper. Hence, we do not see the need for further clarifications on this in the article.

# Peer review: Reviewer response for version 1

**DOI:** 10.21956/openreseurope.14330.r27579

**Published:** 2021-10-06

**Authors:** Marcello Ienca

**Affiliations:** 1 ETH Zurich, Zürich, Switzerland

This article provides an overview of current ethics guidance documents and initiatives for artificial intelligence (AI). The authors argue that the principlist approach which currently dominates the AI ethics discourse is ill-equipped to address the deep socio-political issues and the material impacts of AI such as social inequalities, injustices and environmental damage. Drawing from alternative ethical theories such as the ethics of care and feminist approaches to ethics, the authors propose to complement the existing principled approach with an approach to ethics as attention to context and relations. The article is well informed about the pertinent scientific literature, soundly argued and clearly written. I recommend indexing in its current form.

Is the argument persuasive and supported by appropriate evidence?

Yes

Is the work clearly and cogently presented?

Yes

Is the topic of the essay discussed accurately in the context of the current literature?

Yes

Does the essay contribute to the cultural, historical, social understanding of the field?

Yes

Reviewer Expertise:

Bioethics, neuroethics, ethics of Artificial Intelligence, theoretical cognitive science and the philosophy of technology

I confirm that I have read this submission and believe that I have an appropriate level of expertise to confirm that it is of an acceptable scientific standard.

# Response

**Published:** 2021-11-26

**Authors:** Anais Resseguier

Many thanks for the review and the kind comments on the article.

# Peer review: Reviewer response for version 1

**DOI:** 10.21956/openreseurope.14330.r26881

**Published:** 2021-06-01

**Authors:** Nathalie Grandjean

**Affiliations:** 1 CRIDS (Information Research Center, Law and Society), University of Namur, Namur, Belgium; 2 ESPHIN, University of Namur, Namur, Belgium

Generally I find the article excellent because it highlights an ethics of attention to context that is sorely lacking in AI ethics. However, I would like to address three remarks to the authors, in order to clarify some points that seem obscure or insufficiently developed.

1. I fully agree with the argument of the narratives of digital technologies as immaterial; on the other hand, it seems to me that this immateriality is also at play in the fact that ethical evaluations of AI are formulated in project contexts, i.e. when AI technologies are in the process of being made... which also makes them evanescent, since they are not yet finished.
2. It seems to me that the paper would benefit from better clarification of the difference (both theoretical and practical) between context and materiality; as a reader, it is difficult to understand how the authors formally distinguish between them.
    
3. Finally, the authors mention the concept of "normative capacity" (Canguilhem) but do not mention the concept of "capability" (Sen, Nussbaum), commonly discussed in the literature. It seems to me that this choice should be clarified.

Is the argument persuasive and supported by appropriate evidence?

Yes

Is the work clearly and cogently presented?

Yes

Is the topic of the essay discussed accurately in the context of the current literature?

Yes

Does the essay contribute to the cultural, historical, social understanding of the field?

Yes

Reviewer Expertise:

Ethics, philosophy of technique/technology, gender studies, ethics of care, ethics of attention.

I confirm that I have read this submission and believe that I have an appropriate level of expertise to confirm that it is of an acceptable scientific standard, however I have significant reservations, as outlined above.

# Response

**Published:** 2021-11-26

**Authors:** Anais Resseguier

Thank you very much for the thoughtful review and feedback that helped clarify important aspects of the paper. Regarding the first point, see footnote added on p. 4 that acknowledges the particular context of application of AI ethics review, when there is any, and how this relates to the point raised about the immateriality of AI. Points 2 and 3 have now been clarified. See footnote on p. 6 and endnote 43.
